# John Nkengasong: building African autonomy to support global health

**DOI:** 10.2471/BLT.22.030622

**Published:** 2022-06-01

## Abstract

John Nkengasong talks to Gary Humphreys about setting up the Africa CDC as part of efforts to achieve greater African autonomy in matters of public health.

*Q: How did you become a virologist?*

A: Professor Victor Anomah Ngu, Cameroon’s former public health minister, set me on that path. I had just graduated from university and was considering a career in entomology and he pointed out all the exciting things that were going on in biology and biotechnology. That short meeting changed my life. Later, I did an internship at the medical school in the Department of immunology at the University of Yaoundé, Cameroon, under the late Professor Peter Ndumbe which sharpened my focus on viruses, and I went on to work on hepatitis B as a part of efforts to establish a vaccination programme in Cameroon. That in turn fed into my work on HIV in the late 1980s.

*Q: Inequity of access to antiretrovirals (ARVs) was a defining aspect of the global HIV response. Forty years later access to COVID-19 (coronavirus disease 2019) vaccines is a major concern. Have any lessons been learned?*

A: I am afraid not. In the mid-90s, I was working for the US CDC in Côte d'Ivoire. In 1998 we were part of the very first initiative that UNAIDS (Joint United Nations Programme on HIV/AIDS) established to introduce ARVs in Africa, and other developing countries. Treatment cost around 10 000 [United States] dollars per person per year, and the UN was trying to do something about it. We failed at that time and between 1996 and 2006 the continent witnessed the death of 10 to 12 million people from HIV. History is repeating itself with access to COVID-19 vaccines and therapeutics. I wrote an article in *Nature* about the problem. I wanted to be sure that we as Africans were vocal enough on the topic. But here we are with global vaccination levels in low-income countries running at around 16%. The COVAX initiative was designed to address the equity of access issue and like the Global Fund, represents humankind at its best. Unfortunately, with regards to COVAX, the hope of equitable vaccination has not been fulfilled. COVAX is a promissory note that has not been delivered on. Governments in the position to choose have chosen to prioritize the security needs of their own populations. As a political leader you clearly have a responsibility to those who elected you, but sometimes leadership is required, and I think more could have been done to explain that we are dealing with a pandemic, which is, by definition, global in nature, and requires a global response. I believe countries could have done more to set aside vaccines for use in other parts of the world so that we could vaccinate together and come out of this pandemic together.

“COVAX is a promissory note that has not been delivered on.”

I think the main lesson learned this time, or perhaps re-learned, is that Africa needs to be self-sufficient in diagnostics, vaccines and therapeutics. Not just to become self-sufficient but to be able to contribute to public health development worldwide. The Africa CDC and African Union (AU) have established a target for the continent to try to manufacture at least 60% of total vaccines used in Africa by the year 2040, versus something like 1% of the vaccines being produced now. That does not mean going it alone. Clearly, global cooperation and support will continue to be required to develop the ecosystem and environment needed to achieve our goals.

*Q: Prior to becoming the first director of the Africa CDC, you were working for the US CDC in Atlanta. What made you come back?*

A: Ebola. I came to Addis (Addis Ababa, Ethiopia) in December of 2016 and took up the job in January 2017. If you remember, we had just come out of the Ebola crisis in West Africa. I was very happy at the CDC in the US, but I was getting news of what was happening at home, and I felt I could do something to help. I applied and was fortunate enough to be selected. The US CDC were supportive of the appointment and agreed to second me. It was a great honour, but a great challenge also because we were really starting from zero.

*Q: What do you mean by that?*

A: I mean, for more than a year, I didn't even have an office! I had to move from place to place with my laptop. Eventually, I shared a little table on the 12th floor of the main AU building in Addis Ababa, and then moved into an office with my team. We spent the first two years just building up the institution which included travelling around the continent setting up the five regional centres. It seemed crazy sometimes. Friends asked me why I would take on such a task and joked that I must be going through some sort of mid-life crisis. Whatever it was I know couldn’t have done it without the support of my wife, who had also been doing well at the CDC in Atlanta and literally sacrificed her own career to be by my side in Addis. She’s my hero in this adventure, because she was also a senior level person at the CDC. So, I was working flat out to set up the institution and then the 2019 Ebola outbreak in the DRC (Democratic Republic of the Congo) happened and was closely followed by COVID-19. I remember I was back in Atlanta, spending the end-of-year holidays with my family when news of the Wuhan outbreak started to come through. I was fairly sure that it would only be a matter of time before the virus reached the African continent. I called the Emergency Operations Centre of the Africa CDC in Addis and I said, “Sorry, folks. I know we’re in the middle of the Ebola outbreak, but you need to activate another set of people to focus solely on COVID-19.” It was the beginning of a significant ramping up of resources and today the Africa CDC employs about 319 people.

*Q: Did you get the support you needed from the African Union?*

A: Very much so and I give full credit to the strong leadership of the heads of state, especially President Cyril Ramaphosa who was the chair of the African Union at that time. The AU pushed the envelope for Member States to contribute beyond their own voluntary contribution. I remember a bureau meeting of the AU on the 26th of March 2020 at which President Ramaphosa pledged [US]$ 4 million, [US]$ 2 million of which was to go to the COVID-19 response, and [US]$ 2 million to Africa CDC. It was at that meeting that President Uhuru Kenyatta (President of Kenya), President Paul Kagame (President of Rwanda), and many others stepped up their support. It was like the start of a movement. And foreign contributions for COVID-19 also rose at that time, reaching about [US]$ 200 million, which was matched by the AU, and put into a common basket that we use to support the continent. The Africa CDC acts as secretariat deciding what to do with those funds.

*Q: You saw Africa CDC come to fruition, but are now returning to the United States of America, to head the US President's Emergency Plan for AIDS Relief (PEPFAR). Do you have any misgivings?*

A: Not really. I spent five years building what I call a temple of hope for the African continent and am immensely grateful for the opportunity that was given to me. It has been an extraordinary journey, but a demanding one too and realistically you cannot keep up that level of intensity forever. And I’m still very passionate about HIV, which, as I said, was the focus of my work from a very early stage in my career. We've made significant progress on HIV, but it clearly remains a very serious threat if not managed and recognized as such. And you know that the chief burden of HIV is carried by the African continent. In my new position as PEPFAR head, it is my intention to contribute to really bringing the HIV epidemic under control by the year 2030, which is just around the corner. Key to achieving that goal will be the development of a new vaccine.

“Africa needs to be self-sufficient in diagnostics, vaccines and therapeutics.”

*Q: There has been a considerable investment of resources in developing such a vaccine, without any success. What makes you think it can be achieved?*

A: One of the positive lessons to come out of the COVID-19 pandemic relates to the power of mRNA platform technologies. It is my hope that such technologies can be applied to the development of an HIV vaccine. There are three things that are always critical in disease eradication: effective diagnostics, therapeutics and vaccines. If you miss any of those, you are in trouble. I'm optimistic that the new technologies that have come to the fore in addressing the challenge of COVID-19 may actually have the potential for applications in response to a range of challenges, including HIV but also malaria, tuberculosis and other diseases.

*Q: Are you confident that Africa CDC has the resources it needs going forward?*

A: I think if Africa CDC continues to add value both in Africa and around the world, funding will be sustained. And by that, I mean funding by AU nations but also by partners around the world. On that note I would like to make a direct appeal to the global health community to continue their support, seeing the Africa CDC as a critical component for our collective global health security. For our part, it is essential that we keep the focus on transparency and accountability and tap into the immense resources that we are developing here. I will let history be the judge of what I have achieved here, but personally I am very pleased with the young leaders who are coming through, just as I am pleased with the leadership that is emerging within Africa as a whole. So, when the position of Africa CDC head becomes open, I believe there will be some very strong candidates across Africa who have seen the value of this institution and will want to be part of this exciting adventure in public health.

